# Social inequalities in health and mental health in France. The results of a 2010 population-based survey in Paris Metropolitan Area

**DOI:** 10.1371/journal.pone.0203676

**Published:** 2018-09-14

**Authors:** Elsa Jacquet, Sarah Robert, Pierre Chauvin, Gwenn Menvielle, Maria Melchior, Gladys Ibanez

**Affiliations:** 1 Epidemiology and Population Health Research Centre (CESP), Paris-Sud University, UMRS 1018, Le Kremlin-Bicêtre, France; 2 Department of General Practice, Paris-Sud University, Le Kremlin-Bicêtre, France; 3 Department of Social Epidemiology, Sorbonne University, INSERM, Institut Pierre Louis d'épidémiologie et de Santé Publique (IPLESP UMRS 1136), Paris, France; 4 Department of General Practice, Sorbonne University, Paris, France; Sciensano, BELGIUM

## Abstract

The present study aimed to assess socioeconomic inequalities in general and mental health, depression and substance use disorders (daily tobacco use, hazardous alcohol use). Data from the 2010 SIRS (French acronym for Health, Inequalities, and Social Ruptures) study, which is deemed to be representative of the French-speaking adult population living in the Paris Metropolitan Area, were analysed. Different socioeconomic position indicators were selected: education, income and perceived financial status. Absolute measures (the slope index of inequality (SII)) and relative measures (the odds ratio (OR) and relative index of inequality (RII)) of health inequalities were used. The OR, RII and SII were adjusted for age, household type and migration characteristics and all analyses were performed separately for men and women. The study included 3,006 adults. The results showed significant relative and absolute socioeconomic inequalities in general, mental health and depression for all socioeconomic position indicators considered (education, income, and perceived financial status). The absolute inequalities were greater for women than for men. Strongest inequalities were observed by perceived financial status for men and women. Education seemed to play a stronger role in inequalities for women, whereas, for men, income seemed to play a stronger role. Only few socioeconomic inequalities were found in daily tobacco use, while a reversed gradient was observed for hazardous alcohol use. We hope that these results will be regularly re-evaluated and compared across time in order to monitor socioeconomic inequalities in health.

## Introduction

In 2016, mental and substance use disorders accounted for 18.7% of global years lived with a disability (YLDs). Much greater than the disabilities associated with all infections, all injuries combined, all cardiovascular and circulatory diseases, and all cancers [1]. Socioeconomic circumstances are known to be associated with health and mental health since the end of the 19^th^ century [2]. To date, many studies have documented the association between low socioeconomic position and poor self-rated health, mental health disorders, and substance use disorders [3–10].

Socioeconomic position (SEP) was defined by Mueller and Parcel in 1981 as “the relative position of an individual or family on a hierarchical structure, based on their access to, or control over, wealth, prestige and power” [11]. As SEP relates to health status, Shavers posited that it “is an attempt to capture an individual or group’s access to the basic resources required to achieve and maintain good health” [12]. In epidemiological studies, SEP has mostly been defined by education, income, or occupation [13]. These components may have a direct effect on health, but most likely, they operate through differential exposure to conditions that have more immediate effects on health [13]. Pathways by which SEP influences health include biological determinants, environmental exposure, social environment, health care, behaviour and lifestyle [10].

Several authors have argued that different socioeconomic position indicators implicate different pathways and may relate to (at least partly) different causal processes [14–16]. For example, Macintyre et al. showed that socioeconomic variations in self-rated health and depression depend on the indicator of SEP, and on gender [17]. Education can be taken as a marker of childhood social environment and health literacy. Income can translate into material or immaterial resources for health (better housing, clothing, food, etc) [18,19]. Poorer coping styles, more stressful life events, and weaker social support are some examples of risk factors prevalent when SEP is less favourable, and they could make up a subjective SEP indicator, such as perceived financial status. As SEP indicators are not interchangeable, some authors suggest that several SEP indicators should be examined simultaneously [16,20]. Some studies also demonstrated that different risk factors, including SEP, may operate differently for men and women [21,22]. Cullen et al. described the female resilience pattern, in which women may survive relatively better in circumstances of lesser socioeconomic advantage than men [23]. Taylor et al. showed that women’s greater tendency to seek and mobilise social support, especially during times of stress, could be “one of the most robust gender differences in adult human behaviour” [24].

To properly measure socioeconomic inequalities and gradients in health, current guidelines recommend the use of both absolute and relative measures, such as the slope index of inequality (SII) and relative index of inequality (RII) [25–27]. Our study compares different health outcome indicators in relation to various expressions of SEP, in the light of various analytic approaches. We examine self-rated general and mental health, depression, daily tobacco use, and hazardous alcohol use, according to three SEP dimensions (education, equivalised income, and perceived financial status). We hypothesise (a) that socioeconomic inequalities will be found in all health outcomes, (b) that trends of inequalities in health will differ by gender, and (c) that these inequalities will vary according to socioeconomic indicators.

## Materials and methods

### Study sample

Analyses were based on data from the 2010 wave of the SIRS cohort, a representative socio-epidemiological survey of the French-speaking adult population conducted since 2005 in the Paris metropolitan area (population 6.5 millions). The survey employed a stratified, 3-level, random sampling procedure, based on the ‘IRIS’ system of geographical units, classified according to their socioeconomic profile (poor, average, or rich) and the urban renewal policy attached to them (targeted renewal area or not) [28]. First, 50 census blocks, called ‘IRIS’, with about 2000 inhabitants each, were selected, with an over-representation of the poorest neighbourhoods. Then, 60 households were randomly picked from each surveyed IRIS, and one adult was chosen from each household by the birthday method [29,30]. Only French-speaking adults who gave consent were included; minors and those who were not fluent enough in French to answer the questionnaire, too sick to answer, or refused to participate, were excluded. The latter group was replaced by an entirely new sample selection within the same IRIS (i.e., in a new sampled household). Data were collected between October 2009 and March 2010. A questionnaire was administered face-to-face during home visits. The SIRS cohort study is a collaborative project between the French National Institute for Health and Medical Research (INSERM) and the National Centre for Scientific Research (CNRS). The methodology has been described in more detail elsewhere [31,32].

### Ethics

In accordance with European regulation, French observational studies from data obtained without any additional therapy or monitoring procedure did not need the approval of an institutional review board/independent ethics committee before the year 2014 [33]. The SIRS protocol obtained regulatory approval and legal authorisation from two French national authorities (data-protection approval): the *Comité Consultatif sur le Traitement de l’Information en matière de Recherche dans le domaine de la Santé* (CCTIRS) (authorisation number 904251) and the *Commission Nationale de l’Informatique et des Libertés* (CNIL) (authorisation number 05–1024). Study participants provided verbal informed consent. Written consent was not necessary because the survey did not fall under the category of biomedical research (as defined by French law) and did not collect any personal identification data.

### Outcome measures

#### Self-rated general and mental health, depression

Self-rated general health (SRGH) was measured using the question: ‘How is your health in general?’ Self-rated mental health (SRMH) was measured using the question: ‘How is your mental and emotional health in general?’ The participants responded on a 5-point Likert scale. The ratings were dichotomised into ‘very good and good’ health versus ‘average, poor or very poor’ health.

Depression was assessed using the Mini International Neuropsychiatric Interview (MINI) module related to major depression, based on the Diagnostic and Statistical Manual of Mental Disorders-IV and the International Classification of Diseases-10 criteria [14]. The MINI was used in many studies and its validity was well assessed [34–37].

#### Substance use disorders

Daily tobacco use and hazardous alcohol use were used in our study to estimate substance use disorders. Daily tobacco use was assessed based on the question: ‘Do you smoke, even occasionally?’ The answer ‘I smoke everyday (even one cigarette)’ was counted as ‘Yes’ and the other answers ‘I smoke occasionally’, ‘I quit smoking’, ‘I have never smoked’, were counted as ‘No’. Drinking patterns were explored using the Alcohol Use Disorders Identification Test–Consumption questions (AUDIT-C) [38,39]. This is a three-items questionnaire, each of them scored from 0 to 4 points, giving a maximum total score of 12 points. According to their AUDIT-C score, patients were classified as hazardous drinkers or not (AUDIT-C ≥ 4 in men, ≥ 3 in women) [40].

According to the Keppel methodological guidelines, all health or substance use disorders indicators were expressed in terms of adverse events [26].

#### Socioeconomic position indicators

The SEP of participants was measured by their education, income, and perceived financial status. Education was defined as the highest educational attainment achieved by an individual participant and categorised into four standard hierarchic groups: none or primary education (up to approximately 6 years of education), lower secondary education (up to approximately 9 years of schooling), higher secondary education (up to approximately 12 years), and tertiary education (bachelor’s degree or higher). Equivalised income was calculated based on the Organisation for Economic Co-operation and Development (OECD)-modified scale using self-reported post-tax income. The missing data (8.9% of the responses) were imputed according to a regression model including age, level of education, profession, the number of adults and minors in the household. Equivalised income was classified into four categories according to the 2009 French Taxable Income Survey: below poverty line (€950 per month per consumption unit), between poverty line and median income (€950–1,500 per month per consumption unit), between median income and the income of the wealthiest ten percent of the French population (€1,500–3,000 per month per consumption unit), and above €3,000 per month per consumption unit. Participants’ perceived financial status was assessed using the question: ‘How do you describe your financial situation in general?’ The possible answers were ‘comfortable’, ‘OK’, ‘short of money’ and ‘with financial difficulties’.

### Analytical strategy

First, we described the characteristics of the study population. All proportions were weighted to take into account the sampling method and the poststratification adjustment for age and gender, according to the general population census data. Chi-square tests were used to compare proportions between genders.

Then, we compared the inequalities in general and mental health, depression and substance use disorders, according to the SEP indicators. The results were presented with two series of multivariate logistic regression models. In the first series of models, OR, RII and SII were adjusted for age. Age was included as a categorical variable (18–29 years old; 30–49 years old; 50–64 years old; older than 65 years). In the second series of models, the covariates age, migration characteristics (French, French with foreign parents, foreigners), and household type (one-person; couple with or without children; single-parent) were included. The OR represents the chance (odds) of experiencing poor general or mental health or substance use disorders if individuals are in the lowest SEP, with regard to the highest. Then, the RII and the SI (with 95% confidence intervals) were used to measure socioeconomic gradients in health, mental health, and substance use disorders. Both RII and SII were calculated according to the Kunst and Mackenbach method [41]. Ninety-five percent confidence intervals (95%CI) were calculated for SII and RII as follows: 95%*CI* = *S* ± *c* × *SES*, where S is the point estimate for SII or RII, SES is the standard error for S, and c is the critical 5% value from a t distribution with g − 2 df, with g being the number of SEP groups and df the number of degrees of freedom [42,43]. The RII and SII present the advantage of taking into account the social structure of the population, which is the proportion of each category of socioeconomic indicator in the studied population. They use all available data and are not restricted to comparisons of extreme groups, by treating the SEP indicators as a continuous variable. They constitute two different types of measures of socioeconomic inequalities in health: one absolute (SII) and one relative (RII). The SII is the absolute predicted difference in health outcome rates between the theoretical highest and the lowest SEP in the population; it is interpreted as the difference in predicted health rates at the two extremes of the socioeconomic spectrum, and the RII as their ratio. An SII of 0, or an RII of 1 indicates that there is no consistent relationship between health or substance use and the SEP indicator. A high SII or RII value suggests the existence of a socioeconomic gradient in health, and the higher the score the greater the magnitude of the inequity. A negative SII value means that the health outcome is higher at the lowest level of SEP.

All analyses were conducted for men and women separately, since the literature usually reports gender differences regarding factors associated with mental and substance use disorders. Analyses were performed using the statistical software STATA 13.1 [44].

## Results

### Characteristics of the study population

The sample consisted of 1,595 women (53.1%) and 1,411 men (46.9%). The mean age was 45 years with a minimum of 18 and a maximum of 100 years. More than half of the respondents were living with a partner (married or not) and had one child or more. Men and women were comparable in terms of general health; about 20% reported being in poor health. Men had better mental health and were diagnosed less often with a major depressive disorder, than women (6.0% and 10.4%, respectively). Daily tobacco use was more common among men (men: 31.1%; women: 17.0%) as was hazardous alcohol use (men: 48.4%, women: 34.1%). Men had higher education than women. Both genders were comparable in terms of equivalised income. About 17% of the respondents had an income below poverty line, while more than 40% stated being ‘short of money’ or experiencing ‘financial difficulties’. Perceived financial status was worse for women. Table 1 depicts the prevalence of health and substance use disorders according to SEP indicators.

**Table 1:** Weighted prevalence of health and substance use disorders according to socioeconomic position indicators.

			Poor self-rated general health				Poor self-rated mental health				Major depressive disorder				Daily tobacco use				Hazardous alcohol use			
	men	women	men		women		men		women		men		women		men		women		men		Women	
	n	n	%	p	%	p	%	p	%	p	%	p	%	p	%	p	%	p	%	p	%	p
**Education**				<0.001		<0.001		0.036		<0.001		0.291		<0.001		0.196		0.180		0.003		<0.001
Tertiary	795	902	15.4		14.5		14.5		16.9		5.3		6.9		30.7		17.1		53.1		42.6	
Higher secondary	304	303	22.4		26.5		18.9		29.5		5.1		14.0		33.2		18.2		47.0		26.7	
Lower secondary	205	276	27.8		35.5		21.3		30.1		8.7		16.4		34.8		20.3		40.0		20.4	
Primary school or under	107	114	42.4		53.2		25.4		34.3		8.8		14.1		21.0		5.7		33.9		20.0	
**Equivalised income**				0.002		0.002		0.004		<0.001		<0.001		<0.001		0.475		0.783		<0.001		<0.001
≥ €3.000/CU	301	288	19.2		18.9		15.3		22.3		3.4		8.3		25.7		15.2		60.9		53.1	
€1.500–3.000/CU	555	649	15.1		20.9		11.8		16.5		3.6		8.0		32.7		17.2		55.7		39.2	
€950–1.500/CU	322	381	24.5		23.4		22.7		24.6		7.1		10.5		30.1		17.2		38.4		22.8	
<€950/CU	233	276	31.1		32.4		25.1		35.9		13.5		18.0		35.5		18.5		28.7		18.1	
**Perceived financial status**				<0.001		<0.001		<0.001		<0.001		<0.001		<0.001		0.047		<0.001		<0.001		<0.001
Comfortable	309	332	15.3		14.0		9.0		11.4		3.2		4.0		22.1		10.8		63.1		44.5	
It’s OK	512	524	14.5		17.0		13.6		18.3		4.1		8.2		33.5		16.5		47.9		36.3	
Short of money	388	510	26.5		29.0		21.1		26.2		5.2		12.1		34.8		21.9		44.4		29.8	
Financial difficulties	152	174	38.4		44.5		38.3		46.9		20.8		27.4		37.1		21.5		31.6		22.4	

CU, per consumption unit

The inequalities in general and mental health, depression and substance use disorders, according to the SEP indicators are presented in Table 2 (first series of models controlled for age) and Table 3 (second series of models controlled for age, household type and migration characteristics).

**Table 2:** Socioeconomic inequalities in health and substance use disorders according to socioeconomic position indicators: Odds ratio, relative index of inequality and slope index of inequality controlled for age (95% confidence interval).

	MEN			WOMEN		
	ORage (95%CI)	RIIage (95%CI)	SIIage (95%CI)	ORage (95%CI)	RIIage (95%CI)	SIIage (95%CI)
**Poor self-rated general health**						
Education	2.90 (1.80;4.68)	2.67 (1.65;4.33)	21.20 (16.51;25.90)	4.76 (3.10;7.31)	4.18 (2.95;5.92)	34.78 (30.50;39.07)
Equivalised income	3.66 (2.02;6.63)	3.51 (2.07;5.97)	27.77 (22.39;33.16)	3.50 (2.23;5.50)	2.70 (1.86;3.93)	24.46 (20.87;28.06)
Perceived financial status	5.05 (2.75;9.28)	4.64 (2.71;7.94)	35.33 (26.87;43.80)	7.31 (3.75;14.26)	5.27 (3.16;8.79)	44.89 (35.91;52.92)
**Poor self-rated mental health**						
Education	1.94 (1.06;3.54)	1.90 (1.11;3.24)	11.35 (8.15;14.55)	2.23 (1.43;3.47)	2.58 (1.85;3.59)	22.63 (19.71;25.55)
Equivalised income	2.39 (1.28;4.44)	3.09 (1.49;6.42)	20.10 (14.70;25.51)	2.51 (1.61;3.92)	2.73 (1.76;4.24)	24.09 (21.13;27.05)
Perceived financial status	6.75 (3.71;12.28)	5.83 (3.23;10.52)	33.76 (24.53;42.99)	8.02 (4.89;13.16)	5.29 (3.51;7.99)	42.59 (35.20;49.98)
**Major depressive disorder**						
Education	1.70 (0.75;3.88)	1.90 (0.66;5.44)	3.98 (2.50;5.44)	2.37 (1.33;4.23)	3.96 (2.30;6.84)	15.54 (12.61;18.47)
Equivalised income	5.55 (1.76;17.50)	9.26 (2.40;35.79)	14.36 (8.26;20.46)	2.69 (1.57;4.61)	3.19 (1.88;5.40)	12.65 (9.40;15.89)
Perceived financial status	8.86 (2.82;27.85)	11.84 (3.60;38.95)	17.42 (10.63;24.20)	9.50 (4.87;18.54)	8.57 (4.42;16.64)	26.86 (19.32;34.39)
**Daily tobacco use**						
Education	0.79 (0.45;1.39)	1.08 (0.73;1.59)	2.32 (0.85;3.80)	0.41 (0.18;0.94)	1.10 (0.67;1.82)	1.70 (0.13;3.26)
Equivalised income	1.30 (0.75;2.25)	1.13 (0.67;1.90)	3.72 (3.89;3.54)	0.99 (0.60;1.62)	0.96 (0.60;1.53)	-0.64 (-0.65;-0.63)
Perceived financial status	1.70 (0.96;2.99)	1.48 (0.87;2.52)	12.56 (11.01;14.11)	1.97 (1.17;3.30)	2.11 (1.43;3.11)	13.71 (11.06;16.38)
**Hazardous alcohol use**						
Education	0.37 (0.23;0.62)	0.55 (0.39;0.76)	-28.40 (-28.87;-30.01)	0.24 (0.15;0.41)	0.25 (0.19;0.34)	-45.59 (-41.63;-49.55)
Equivalised income	0.27 (0.15;0.47)	0.42 (0.28;0.62)	-42.28 (-38.98;-55.58)	0.20 (0.12;0.34)	0.26 (0.18;0.38)	-44.65 (-40.61;-48.70)
Perceived financial status	0.28 (0.17;0.49)	0.52 (0.38;0.70)	-31.29 (-30.23;-32.35)	0.37 (0.24;0.59)	0.49 (0.33;0.71)	-23.68 (-19.10;-28.27)

ORage, odds ratio controlled for age; CI, confidence interval; RIIage, relative index of inequality controlled for age; SIIage, slope index of inequality controlled for age

**Table 3:** Socioeconomic inequalities in health and substance use disorders according to socioeconomic position indicators: Odds ratio, relative index of inequality and slope index of inequality controlled for age, household type and migration characteristics (95% confidence interval).

	MEN			WOMEN		
	OR (95%CI)	RII (95%CI)	SII (95%CI)	OR (95%CI)	RII (95%CI)	SII (95%CI)
**Poor self-rated general health**						
Education	2.09 (1.23;3.55)	2.14 (1.34;3.41)	16.07 (12.99;19.16)	4.28 (2.75;6.68)	3.60 (2.59;5.00)	30.53 (27.07;34.00)
Equivalised income	3.14 (1.60;6.16)	2.87 (1.69;5.15)	22.72 (17.73;27.70)	2.71 (1.69;4.34)	2.15 (1.44;3.22)	18.37 (15.72;21.00)
Perceived financial status	4;01 (2.11;7.62)	3.61 (2.09;6.24)	28.39 (21.60;35.18)	6.30 (3.13;12.69)	4.56 (2.63;7.89)	39.44 (31.05;47.85)
**Poor self-rated mental health**						
Education	1.79 (0.89;3.58)	1.81 (0.98;3.34)	10.49 (7.03;13.94)	2.06 (1.31;3.26)	2.30 (1.66;3.21)	19.66 (17.53;21.81)
Equivalised income	2.43 (1.28;4.61)	2.95 (1.45;6.00)	19.15 (13.95;24.36)	2.10 (1.29;3.42)	2.26 (1.39;3.67)	19.07 (16.53;21.61)
Perceived financial status	6.34 (3.35;12;00)	5.41 (3.00;9.73)	31.94 (22.48;41.39)	6.72 (3.93;11.50)	4.52 (2.88;7.10)	37.48 (29.87;45.07)
**Major depressive disorder**						
Education	1.44 (0.57;3.62)	1.67 (0.54;5.16)	3.15 (1.93;4.37)	2.18 (1.20;3.96)	3.52 (1.96;6.29)	13.92 (10.94;16.90)
Equivalised income	5.51 (1.76;17.27)	8.50 (2.11;34.22)	13.58 (7.01;25.14)	2.22 (1.24;3.96)	2.53 (1.42;4.49)	9.84 (7.06;12.63)
Perceived financial status	7.85 (2.50;24.70)	10.00 (3.43;29.03)	15.63 (9.73;18.53)	8.14 (4.02;16.47)	7.21 (3.68;14.13)	23.79 (16.95;30.62)
**Daily tobacco use**						
Education	1.12 (0.61;2.05)	1.30 (0.88;1.92)	8.35 (5.89;10.81)	0.52 (0.23;1.18)	1.28 (0.74;2.23)	4.36 (1.29;7.42)
Equivalised income	1.58 (0.90;2.79)	1.29 (0.73;2.26)	7.90 (6.67;9.12)	0.95 (0.54;1.67)	0.91 (0.54;1.55)	-1.57 (-1.93;-1.21)
Perceived financial status	1.93 (1.04;3.56)	1.65 (0.94;2.92)	16.23 (13.01;19.45)	1.66 (0.95;2.91)	1.90 (1.26;2.87)	11.63 (8.85;14.40)
**Hazardous alcohol use**						
Education	0.47 (0.28;0.82)	0.64 (0.44;0.92)	-21.09 (-18.62;-23.57)	0.29 (0.17;0.50)	0.29 (0.22;0.40)	-40,00 (-36.41;-43.54)
Equivalised income	0.33 (0.19;0.59)	0.49 (0.32;0.75)	-33.54 (-30.02;-37.07)	0.22 (0.12;0.40)	0.28 (0.19;0.42)	-41.12 (-36.53;-45.72)
Perceived financial status	0.38 (0.21;0.66)	0.62 (0.45;0.86)	-22.63 (-21.27;-23.99)	0.36 (0.22;0.57)	0.49 (0.35;0.70)	-23.22 (-18.52;-27.93)

OR, odds ratio; CI, confidence interval; RII, relative index of inequality; SII, slope index of inequality

### General, mental health and depression

The results of relative measurements showed socioeconomic inequalities for most of the considered SEP indicators (education, income, and perceived financial status). There was a higher risk of both poor general and mental health for the most disadvantaged SEP, compared to the most advantaged (OR) and an inequality gradient (RII) in general and mental health.

Absolute inequalities for general and mental health varied widely according to SEP indicators. In adjusted analyses, the SII ranged from 3.15% [95%CI 1.93 to 4.37], to 39.44% [95%CI 31.05 to 47.85], suggesting that poor general health was estimated to be up to 39.44% points higher at the bottom, versus the top of perceived financial status distribution for women.

The magnitude of educational inequality among women was statistically higher than men. For example, the SII_poor_SRGH_ was 30.53 [95%CI 27.07 to 34.00] for women versus 16.07 [95%CI 12.99 to 19.16] for men. Similar results were found for poor mental health and depression. For all analyses, strongest inequalities were observed by perceived financial status. Education seemed to play a stronger role in inequalities for women, whereas, for men, income seemed to play a stronger role.

### Substance use disorders

For daily tobacco use, the results of relative measurements did not show socioeconomic inequalities, with most of the SEP indicators considered, except for the perceived financial status. Absolute inequalities for daily tobacco use varied according to SEP indicators. In adjusted analyses, the SII_men_ ranged from 7.90% for the subgroup ‘education’ [95%CI 6.67 to 9.12] to 16.23% for the subgroup ‘perceived financial status’ [95%CI 13.01 to 19.45]. Results were similar for women, considering education and perceived financial status. For all analyses, strongest inequalities were observed by perceived financial status.

For hazardous alcohol use, the results of relative measurements showed socioeconomic inequalities for all the SEP indicators considered (education, income, perceived financial status). There was a higher risk of hazardous alcohol use for the most advantaged SEP, compared to the most disadvantaged (OR) and a reverse inequality gradient (RII). In adjusted analyses, the SII_men_ ranged from -21.09% for the subgroup ‘education’ [95%CI -18.62 to -23.57] to -33.54% for the subgroup ‘equivalised income’ [95%CI -30.02 to -37.07]. These inequalities were greater for women than for men. For all analyses, strongest inequalities were observed by equivalised income.

## Discussion

### Summary of findings

The results showed significant relative and absolute socioeconomic inequalities in general, mental health and depression for all considered SEP indicators (education, income, and perceived financial status). The absolute inequalities were greater for women than for men. Strongest inequalities were observed by perceived financial status for men and women. Education seemed to play a stronger role in inequalities for women, whereas for men, income seemed to play a stronger role. Few socioeconomic inequalities were found in daily tobacco use, while a reversed gradient was observed for hazardous alcohol use. These results indicate that our hypotheses seem to be confirmed.

### Comparison with other studies

Many studies have demonstrated the existence of socioeconomic inequalities in general health and mental health [45–47]. Our results show similar associations in France, using income, education and perceived financial status. In our study, we observed larger absolute inequalities in women than in men. These differences can result from a higher prevalence in women of self-rated health, mental health and depression, compared to men. This result confirms the need to present both relative and absolute results in further studies, to get a comprehensive picture of inequalities.

The higher prevalence of mental health disorders in lower socioeconomic groups is likely to be explained by causation and selection processes [47]. Persons in a lower socio-economic position may experience mental health disorders (causation), which may lead to a downward SEP (selection). In the causation process, mental health inequalities are mainly caused by the higher exposure of lower socioeconomic groups to unfavourable material, psychosocial and behavioural factors. In our study, the strongest inequalities were observed by perceived financial status. This result could support the relative deprivation hypothesis, which “posits that increasing income inequality in a society will heighten an individual’s sense of relative deprivation, resulting in frustration, shame, stress, and maladaptive coping responses (e.g. smoking)” [48,49]. We hypothesise that perceived financial status reflects the current economic burden and stress level of an individual, more than education or income. Our study also shows that education seemed to play a stronger role in inequalities for women, whereas, for men, income seemed to play a stronger role. These associations have been little studied and could be further investigated in cohort studies [50–52].

Our results show absolute inequalities for daily tobacco use. This inequality is well known, especially in western European countries [47,53]. However, we did not observe relative inequalities for daily tobacco use. This result is due to the fact that prevalence of daily tobacco use among “primary school or under” respondents was very low in our study. If we restrict the analysis to people with at least secondary education, a similar gradient consistent with the literature is observed for relative and absolute inequalities. Our results also describe a reverse association between SEP (assessed by education, income and perceived financial status) and hazardous alcohol use. A European study demonstrated that in France (as well as in Germany, Switzerland, Austria, and the Netherlands), women with the highest education level were more likely to have high alcohol consumption habits [54]. The Paris Metropolitan Area is inhabited by large migrant groups, many of whom are Muslims, who generally do not drink alcohol. However, the reverse association between hazardous alcohol use and financial SEP indicators remained significant when controlled for migration characteristics. The existing studies in France revealed that daily alcohol use is generally associated with positive beliefs and expectations, especially for older people [55]. This may partly account for the positive association between higher SEP and higher alcohol consumption. Our results are likely to differ for heavy drinking and should be further investigated.

### Strengths and limitations

To our knowledge, this is the first study in France to have systematically evaluated socioeconomic inequalities and gradients in general health, mental health, and substance use disorders. The SIRS survey was representative of the population residing in the Paris Metropolitan Area (Paris and its neighbouring departments). Data collection through face-to-face interviews served to confirm certain data and limit the amount of missing data. According to recent guidelines, multiple measures of socioeconomic inequalities in health were used. The ORs allowed us to estimate an odds at a given point in time, and to compare our results with numerous studies. The advantage of RII is that it can adapt to the structural changes of a population in time or space, and compare men and women, or different territories, with different socioeconomic structures. The use of summary measures of inequalities, such as RII and SII, involved assessing SEPs with hierarchical indicators. We used income and education, which are among the most commonly used indicators, and perceived financial status, which is known to be a determinant of self-rated psychological well-being [56]. Comparisons between socioeconomic groups were performed by examining the overlap between confidence intervals. No statistical test was undertaken, though the p-value may provide complementary types of information [57,58]. The cohort applied only to French-speaking residents in the Paris Metropolitan Area. It excluded the non-French-speaking migrant population living in precarious conditions, and potentially in poorer health. Likewise, homeless people were not surveyed. This might have resulted in undervalued social inequalities [59]. Our results were limited to our sample size and the statistical power of our analysis, especially for depression, since there were only 85 men and 166 women diagnosed with depression in our study population. This can explain the large confidence intervals of OR and RII, regarding socioeconomic inequalities in depression among men. Finally, we chose not to examine drug use due to the very low positive response rate to the question of drug consumption in the SIRS study.

## Conclusion

The current persistence of socioeconomic inequalities in health, despite public health policies and campaigns, makes it important to provide data to quantify and monitor socioeconomic inequalities in several fields of health. Our study provided this type of data and proved that for individuals with the least favourable conditions, socioeconomic inequalities in general and mental health are marked. Our results, which could be monitored over time and compared across countries, are policy relevant. We hope that this work could help improve programs targeting disadvantaged subgroups in general health, mental health, and substance use disorders.

## Supporting information

S1 Appendix English questionnaire. (PDF)

S2 Appendix French questionnaire. (PDF)
