# Supplementary material for: Social inequalities in health and mental health in France. The results of a 2010 population-based survey in Paris Metropolitan Area
Source: PLoS One. 2018 Sep 14;13(9):e0203676. doi: 10.1371/journal.pone.0203676 (PMC6138404; doi:10.1371/journal.pone.0203676)
Supplement: S1 Appendix — (PDF) [file pone.0203676.s001.pdf]

Inserm

Institut national
de la santé et de la recherche médicale

cnrs dépasser les frontières

Questionnaire – SIRS 2010

Date	

Num Ident	

If the person responded to the 2007 survey, tick "Yes" ☐ Yes ☐ No

1. How is your health in general?

☐ very good

☐ good

☐ medium

☐ poor

☐ very poor

2. How is your mental and emotional health in general?

☐ very good

☐ good

☐ medium

☐ poor

☐ very poor

3. Mini International Neuropsychiatric Interview

Have you been consistently depressed or down, most of the day, nearly every day, for the past two weeks? ☐ Yes ☐ No

In the past two weeks, have you been much less interested in most things or much less able to enjoy the things you used to enjoy most of the time? ☐ Yes ☐ No

Over the past two weeks, when you felt depressed or uninterested:

Was your appetite decreased or increased nearly every day? Did your weight decrease or increase without trying intentionally (i.e., by ±5% of body weight or ±8 lbs. or ±3.5 kgs., for a 160 lb./70 kg. person in a month)? ☐ Yes ☐ No

Did you have trouble sleeping nearly every night (difficulty falling asleep, waking up in the middle of the night, early morning wakening or sleeping excessively)? ☐ Yes ☐ No

Did you talk or move more slowly than normal or were you fidgety, restless or having trouble sitting still almost every day? ☐ Yes ☐ No

Did you feel tired or without energy almost every day? ☐ Yes ☐ No

Did you feel worthless or guilty almost every day? ☐ Yes ☐ No

Did you have difficulty concentrating or making decisions almost every day?

☐ Yes ☐ No

Did you repeatedly consider hurting yourself, feel suicidal, or wish that you were dead?

☐ Yes ☐ No

4. Do you smoke, even occasionally?

☐ I smoke everyday (even one cigarette)

☐ I smoke occasionally

☐ I quit smoking

☐ I have never smoked

5. How often do you have a drink containing alcohol?

☐ Never

☐ Monthly or less

☐ 2-4 times a month

☐ 2-3 times a week

☐ 4 or more times a week

6. How many standard drinks containing alcohol do you have on a typical day?

!! Instructions: we talk about "standard glasses", show cardboard

☐ 1 or 2

☐ 3 or 4

☐ 5 or 6

☐ 7 to 9

☐ 10 or more

7. How often do you have six or more drinks on one occasion?

☐ Never

☐ Less than monthly

☐ Monthly

☐ Weekly

☐ Daily or almost daily

8. How much money do you (or your family) have to live monthly, in euros?

☐ Amount:

☐ Do not know

☐ Do not want to answer

9. How do you describe your financial situation in general?

☐ Comfortable

☐ Okay

☐ Short of money

☐ Financial difficulties

10. What is your highest educational attainment?

☐ None

☐ Primary education (up to approximately 6 years of education)

☐ Lower secondary education (up to approximately 9 years of schooling)

☐ Higher secondary education (up to approximately 11 years)

☐ Tertiary education (bachelor's degree or higher)

11. What is the highest degree you have obtained?

☐ None

☐ Certificate of primary school

☐ First secondary school diploma

☐ Baccalaureate

☐ Baccalaureate + 2

☐ Baccalaureate + 3 or more

12. What is your year of birth?

☐ ☐ ☐ ☐

13. What is your household type?

☐ One-person household

☐ Couple without children

☐ Couple with others

☐ Several couples

☐ One parent with 1 child or more (less than 30 years old)

☐ One parent with 1 child or more (less than 30 years old), with others

☐ Many persons (>30 years) not living as a couple

14. What is (or was) the nationality of your father?

☐ French

☐ Other

☐ Do not know

15. What is (or was) the nationality of your mother?

☐ French

☐ Other

☐ Do not know

16. Currently, what is your nationality?

☐ French

☐ Other

☐ Do not know

17. Can you list all the people who usually reside in your dwelling?

1. ☐ Man ☐ Woman Age ☐☐☐ years old
2. ☐ Man ☐ Woman Age ☐☐☐ years old
3. ☐ Man ☐ Woman Age ☐☐☐ years old
4. ☐ Man ☐ Woman Age ☐☐☐ years old
5. ☐ Man ☐ Woman Age ☐☐☐ years old
6. ☐ Man ☐ Woman Age ☐☐☐ years old
7. ☐ Man ☐ Woman Age ☐☐☐ years old
8. ☐ Man ☐ Woman Age ☐☐☐ years old
9. ☐ Man ☐ Woman Age ☐☐☐ years old
