# Supplementary material for: Social inequalities in health and mental health in France. The results of a 2010 population-based survey in Paris Metropolitan Area
Source: PLoS One. 2018 Sep 14;13(9):e0203676. doi: 10.1371/journal.pone.0203676 (PMC6138404; doi:10.1371/journal.pone.0203676)
Supplement: S2 Appendix — (PDF) [file pone.0203676.s002.pdf]

Inserm

Institut national
de la santé et de la recherche médicale

cnrs dépasser les frontières

Questionnaire – SIRS 2010

Date	

Num Ident	

Si l'enquêté a répondu à la vague de 2007 cocher « Oui » ☐ Yes ☐ No

1. Comment est votre état de santé général ?

☐ très bon

☐ bon

☐ moyen

☐ mauvais

☐ très mauvais

2. Comment est votre état de santé psychologique et émotionnel ?

☐ très bon

☐ bon

☐ moyen

☐ mauvais

☐ très mauvais

3. Mini International Neuropsychiatric Interview

Au cours des deux derrières semaines,

• Vous êtes-vous senti particulièrement triste, cafardeux, déprimé, la plupart du temps au cours de la journée, et ce, presque tous les jours ? ☐ Yes ☐ No

• Aviez-vous presque tout le temps le sentiment de n'avoir plus goût à rien, d'avoir perdu l'intérêt ou le plaisir pour les choses qui vous plaisent habituellement ? ☐ Yes ☐ No

• Vous sentiez-vous presque tout le temps fatigué, sans énergie ? ☐ Yes ☐ No

Si au moins 2 « oui » :

Au cours de ces deux dernières semaines, lorsque vous vous sentiez "déprimé" et / ou "sans intérêt pour la plupart des choses" et / ou "fatigué" :

• Votre appétit a-t-il notablement changé ou avez-vous pris ou perdu du poids sans en avoir l'intention ? ☐ Yes ☐ No

• Aviez-vous des problèmes de sommeil (endormissement, réveils nocturnes ou précoces, hypersomnie) presque toutes les nuits ? ☐ Yes ☐ No

• Parliez-vous ou vous déplaciez-vous plus lentement que d'habitude, ou au contraire vous sentiez-vous agité(e) et aviez-vous du mal à rester en place ? ☐ Yes ☐ No

• Manquiez-vous de confiance en vous-même, ou vous sentiez-vous sans valeur, voire inférieur(e) aux autres ? ☐ Yes ☐ No

• Vous êtes-vous fait des reproches, ou vous êtes-vous senti(e) coupable ? ☐ Yes ☐ No

• Avez-vous eu du mal à réfléchir ou à vous concentrer, ou aviez-vous du mal à prendre des décisions ? ☐ Yes ☐ No

• Avez-vous eu à plusieurs reprises des idées noires comme penser qu'il vaudrait mieux que vous soyez mort(e), ou avez-vous pensé à vous faire du mal ? ☐ Yes ☐ No

4. Est-ce que vous fumez, ne serait-ce que de temps en temps ?

☐ Vous fumez quotidiennement (au moins une cigarette par jour)

☐ Vous fumez occasionnellement

☐ Vous ne fumez plus

☐ Vous n'avez jamais fumé

5. Combien de fois vous arrive-t-il de consommer de l'alcool ? ☐ Never

☐ 1 fois par mois ou moins

☐ 2-4 fois par mois

☐ 2-3 fois par semaine

☐ 4 fois ou plus par semaine

6. Au cours d'une journée ordinaire où vous buvez de l'alcool, combien de verres d'alcool buvez-vous, que ce soit de la bière, du vin ou tout autre type d'alcool ?

!! Consigne : on parle de verres standard, Montrer carton

☐ 1 ou 2

☐ 3 ou 4

☐ 5 ou 6

☐ 7 à 9

☐ 10 ou plus

7. Au cours d'une même occasion / moment, combien de fois vous arrive-t-il de boire six verres standard ou plus ?

☐ Jamais

☐ Moins d'une fois par mois

☐ Une fois par mois

☐ Une fois par semaine

☐ Chaque jour ou presque ?

8. Au total votre ménage dispose par mois de approximativement :

☐ En clair, euros ☐ En tranche, montrer la carte

☐ Ne sait pas

☐ Ne veut pas répondre

9. Etant donnés les revenus de votre ménage, actuellement, diriez-vous que financièrement

☐ Vous êtes à l'aise

☐ Ca va

☐ C'est juste, il faut faire attention

☐ Vous y arrivez difficilement

10. Quel est votre niveau d'étude ?

☐ Jamais scolarisé

☐ Enseignement primaire

☐ Enseignement secondaire 1er cycle

☐ Enseignement secondaire 2ème cycle

☐ Enseignement supérieur

11. Quel est le diplôme le plus élevé que vous avez obtenu ?

☐ Aucun diplôme

☐ CEP (certificat d'études primaires)

☐ BEPC, brevet élémentaire (BE), BEPS, CAP, BEP ou autre diplôme de ce niveau

☐ Baccalaureat

☐ Baccalaureat + 2 ans

☐ Baccalaureat + 3 ans ou plus

12. Quelle est votre année de naissance?

☐ ☐ ☐ ☐

13. Quelle est votre type de ménage?

☐ ménage d'une seule personne

☐ ménage mononucléaire : 1 couple avec ou sans enfant

☐ ménage mononucléaire élargi : 1 couple (avec ou sans enfant) avec d'autres personnes

☐ ménage pluri-nucléaire (élargi ou non) : plusieurs couples (avec ou sans enfants)

☐ famille monoparentale : 1 parent avec enfant(s) de moins de 30 ans

☐ famille monoparentale élargie : 1 parent avec enfant(s) avec d'autres personnes

☐ ménage d'"isolés" : plusieurs personnes ne vivant pas en couple (y compris un parent avec un enfant de plus de 30 ans)

☐ Autre

---

14. Quelle est (ou était) la nationalité de votre père ?

☐ Française

☐ Autre

☐ Ne sait pas

---

15. Quelle est (ou était) la nationalité de votre mère?

☐ Française

☐ Autre

☐ Ne sait pas

---

16. Aujourd'hui, quelle est votre nationalité ?

☐ Française

☐ Autre

☐ Ne sait pas

---

17. Pouvez-vous énumérer toutes les personnes qui résident habituellement dans votre logement, en précisant leur prénom et quelques caractéristiques.?

1. ☐ Homme ☐ Femme Age ☐☐☐ ans
2. ☐ Homme ☐ Femme Age ☐☐☐ ans
3. ☐ Homme ☐ Femme Age ☐☐☐ ans
4. ☐ Homme ☐ Femme Age ☐☐☐ ans
5. ☐ Homme ☐ Femme Age ☐☐☐ ans
6. ☐ Homme ☐ Femme Age ☐☐☐ ans
7. ☐ Homme ☐ Femme Age ☐☐☐ ans
8. ☐ Homme ☐ Femme Age ☐☐☐ ans
9. ☐ Homme ☐ Femme Age ☐☐☐ ans
